# EPIDEMIOLOGICAL PROFILE AND EVOLUTION IN MUSCULOSKELETAL TUMORS AT THE LEVEL OF THE ELBOW

**DOI:** 10.1590/1413-785220233101e261309

**Published:** 2023-02-20

**Authors:** VINICIUS DE ABREU MAZZOLIN, JULIA ROCHA KALLUF, FIAMA KURODA OGATA, NATHALIA SUNDIN PALMEIRA DE OLIVEIRA, JAIRO GRECO GARCIA, MARCELO DE TOLEDO PETRILLI, MARCOS KORUKIAN, DAN CARAI MAIA VIOLA

**Affiliations:** 1 Grupo de Apoio à Criança com Câncer, São José dos Campos, SP, Brazil.; 2 Universidade Federal de São Paulo, Paulista School of Medicine, Department of Orthopedics and Traumatology, São Paulo, SP, Brazil.; 3 Universidade do Estado do Rio de Janeiro, Pedro Ernesto University Hospital, Orthopedics and Traumatology Education and Care Unit, Rio de Janeiro, RJ, Brazil.; 4 Grupo de Apoio ao Adolescente e à Criança com Câncer, Institute of Pediatrics Oncology, São Paulo, SP, Brazil.; 5 Columbia University Medical Center, New York, NY, USA.

**Keywords:** Neoplasms, Sarcoma, Elbow, Amputation, Tumor Local Recurrence, Neoplasias, Sarcoma, Cotovelo, Amputação, Recidiva Local de Neoplasia

## Abstract

**Objective:**

To present the epidemiological profile of bone and soft tissue tumors that affect the elbow region treated at an oncology referral center in Brazil.

**Methods:**

Retrospective observational case series study to evaluate the results of elbow cancer undergoing clinical and/or surgical treatment with the first visit from 1990 to 2020. The dependent variables were benign bone tumor, malignant bone tumor, benign soft tissue tumor, malignant soft tissue tumor. Independent variables were sex, age; presence of symptoms (pain/increase in local volume/fracture); diagnosis; treatment and recurrence.

**Results:**

In total, 37 patients were included, 51.35% of whom were female, with a mean age at diagnosis of 33.5 years. Soft tissue neoplasms correspond to 51% of cases against 49% of bone tumors. Among the symptoms, the general prevalence of pain was 56.75%, the general increase in local volume occurred in 54.04% of the patients and the presence of fractures in 13.43%. Surgical treatment occurred in 75.67% of cases and recurrence in 16.21% of cases.

**Conclusion:**

The tumors that affect the elbow in our series correspond mostly to benign tumors, involving bone or soft tissues, with a higher occurrence in young adult patients. ***Level of Evidence IV, Case Series.***

## RESUMO

**Objetivo:**

Apresentar o perfil epidemiológico dos tumores ósseos e de partes moles que acometem a região do cotovelo.

**Métodos:**

Estudo observacional retrospectivo de série de casos para avaliação dos resultados de neoplasia do cotovelo submetidos a tratamento clínico e/ou cirúrgico cujo primeiro atendimento se deu entre 1990 e 2020. As variáveis dependentes foram: tumor ósseo benigno, tumor ósseo maligno, tumor de partes moles benigno, tumor de partes moles maligno. A variáveis independentes foram: sexo; idade; presença de sintomas (dor, aumento de volume local, fratura); lateralidade; diagnóstico; tratamento; e recidiva.

**Resultados:**

Foram incluídos 37 pacientes, sendo 51,35% do sexo feminino, com média de idade ao diagnóstico de 33,5 anos. As neoplasias de partes moles correspondem a 51% dos casos contra 49% de tumores ósseo. Dentre os sintomas a prevalência geral de dor foi de 56,75%, foi observado o aumento geral de volume local em 54,04% pacientes e a presença de fraturas em 13,43%. O tratamento cirúrgico ocorreu em 75,67% dos casos e a recidiva em 16,21%.

**Conclusão:**

Nesta série, os tumores que acometem o cotovelo são majoritariamente tumores benignos, de acometimento ósseo ou de partes moles, com maior ocorrência em pacientes adultos jovens. **Nível de Evidência IV, Série de Casos.**

## INTRODUCTION

Bone tumors, malignant or benign, are rare lesions compared to other neoplasms, and the involvement of the elbow region is even less common, corresponding to about 1% of bone neoplasms.^1^ Although bone and soft tissue tumors of the elbow and forearm are rare, the general orthopedist should be aware that these tumors may occur and should be prepared to treat them properly.^2^

Most available texts in the medical literature on neoplastic elbow lesions are related to case reports. Few articles address the incidence of bone neoplasms in this region due to their rarity; but they generally show similar data reporting the distal end of the humerus as the part of highest incidence,^1^ ^),(^ ^3^ and corresponding to benign neoplasms.^3^ ^),(^ ^4^ To date, the literature only has two case series review studies, with groups of 25^3^ and 75^1^ patients.

The study of the elbow is particularly important due to the anatomical complexity of the main neurovascular structures that are close to each other (median nerve and brachial artery with its division into ulnar artery and radial artery in the anterior part, ulnar nerve at the medial edge, and radial nerve at the lateral edge) and the small amount of soft tissue, especially in the posterior part. This may hinder resection with a wide oncological margin, as these neurovascular structures and coverage and reconstruction options may be affected.^5^ Moreover, tumors in the elbow region usually have higher rates of residual disease and, thus, a higher incidence of local recurrence.^5^

Differential diagnosis of a patient with bone or soft tissue mass includes infection, neoplasia, trauma, and inflammatory processes; pain is the most common but non-discriminatory symptom. With a good anamnesis, careful physical examination and aid of imaging tests, such as plain radiographs, diagnosis can usually be made or at least the possibilities may be limited to specific conditions.^5^

Magnetic resonance imaging is essential for local evaluation of the lesion, size, characteristics of neoplastic tissue, and involvement of close neurovascular structures. Other diagnostic modalities, such as computed tomography and scintigraphy are performed only in cases of lesions with aggressive characteristics. A histological sample should be obtained in aggressive bone lesions to imaging tests and in those whose diagnosis is unclear and is essential for treatment, usually by bone biopsy needle, and can be performed under radioscopy, tomography, or ultrasound.^6^ An inadequate or inaccurate biopsy may lead to poor results regarding limb recovery and patient survival.^7^ ^),(^ ^8^

This study aimed to characterize tumors in the elbow that presented the epidemiological profile of these lesions treated in an oncology reference center in Brazil.

## MATERIAL AND METHODS

A retrospective observational study of case series was conducted to evaluate the results of musculoskeletal tumors in the elbow. Data were collected from medical and imaging records of patients, and a specific database was built for this study with total protection regarding the identification of patients. The study was approved by the Institutional Ethics Committee and is registered on Plataforma Brasil under number 41308720.0.0000.5505.

Medical records of 37 patients diagnosed with elbow neoplasia subjected to clinical and/or surgical treatment with the first service care from 1990 to 2020 were analyzed.

The inclusion criteria were patients of both sexes, with no age limit, monitored in the institution, with musculoskeletal neoplasia in the elbow region, defined as: the region between the medial and lateral epicondyle of the distal humerus, capitulum, head of the radius, and olecranon.

- Bone injury in the distal humerus at the upper limit of Heim’s square including medial epicondyles and articular surface;
- Bone injury of the proximal end of the radius, defined by the metaphyseal region (upper limit of Heim’s square) to the articular surface;
- Bone injury in the olecranon;
- Soft tissue lesions covering the distal anatomical regions to the upper limit of the metaphyseal region of the distal humerus and proximal to the distal limit of the metaphyseal region of the head of the radius.

Lesions that made a differential diagnosis with musculoskeletal neoplasms, such as musculoskeletal infections (osteomyelitis, soft tissue infection) of this region, were excluded.

All patients with tumors of proximal or distal upper limbs to the area of interest or lesions with contiguity extension to the elbow, but whose epicenter was not in the studied region were excluded. The lack of patient compliance to participate in the study, at any time, was also considered an exclusion criterion.

All patients were evaluated according to general epidemiological variables: (1) sex; (2) age; (3) presence of symptoms (pain/increase in local volume/fracture), (4) laterality; (5) diagnosis; (6) treatment; (7) recurrence of the lesion after surgical treatment. This study considered the following elbow lesions a dependent variable: Benign Bone Tumor, Malignant Bone Tumor, Benign Soft Tissue Tumor, and Malignant Soft Tissue Tumor.

The studied variables were collected by analysis of medical records. Bone tumors were confirmed by biopsy.

The construction of the database and graph creation was performed using the Excel (Microsoft^®^) software. The SPSS^®^ software (IBM, V21) was used for statistical analysis. Descriptive analyses are presented in absolute number (n) and relative frequency (%), mean, and standard deviation. Fisher’s exact test was used to compare relative frequencies lower than five. ANOVA was used to compare the means of numerical variables of three or groups. Epidemiological analyses of the studied variables were performed, describing categorical and continuous variables.

## RESULTS

The study included 37 patients with tumor lesions in the elbow region, of which 19 (51.35%) were female and 18 (48.64%) were male. Age ranged from 3 to 77 years, with a mean of 33.5 ± 21.92 years. Right-sided involvement occurred in 51.35% (n = 19) and left-sided involvement in 48.64% (n = 18). Among the most common symptoms, pain complaints were present in 21 (56.75%) patients, local volume increase occurred in 20 (54.05%), and fractures in six (16.21%). Surgical intervention was performed in 28 patients (75.67%). Recurrence was diagnosed in 16.21% (n = 6) of the cases (Table 1).

**Table 1:** Sample characterization (n = 37).

	Total	BBT	MBT	Benign STT	Malignant STT
	n (%)	n (%)	n (%)	n (%)	n (%)
		**12 (32.43%)**	**6 (16.21%)**	10 (27.02%)	9 (24.32%)
**Sex**					
Female	19 (51.35%)	5 (41.66%)	3 (50%)	9 (90%)	2 (22.22%)
Male	18 (48.64%)	7 (58.33%)	3 (50%)	1 (10%)	7 (77.77%)
**Age** (mean/SD)	33.5 ± 21.92	17.08 ± 10.58	33.25 ± 26.5	46.56 ± 19.63	42.44 ± 18.93
**Affected side**					
Left	18 (48.64%)	6 (50.55%)	5 (83.33%)	4 (40%)	3 (33.33%)
Right	19 (51.35%)	6 (50%)	1 (16.66%)	6 (60%)	6 (66.67%)
**Pain**					
No	16 (43.24%)	5 (41.66%)	4 (66.66%)	3 (30%)	4 (44.44%)
Yes	21 (56.75%)	7 (58.33%)	2 (33.33%)	7 (70%)	5 (55.55%)
**Local volume increase**					
No	17 (45.94%)	8 (66.66%)	6 (100%)	2 (20%)	1 (11.11%)
Yes	20 (54.04%)	4 (33.33%)	0 (0%)	8 (80%)	8 (88.88%)
**Fracture**					
No	31 (83.78%)	9 (75%)	4 (66.67%)	10 (100%)	9 (88.88%)
Yes	6 (16.21%)	3 (25%)	2 (33.33%)	0 (0%)	1 (11.11%)
**Surgical treatment**					
No	9 (24.32%)	5 (41.66%)	0 (0%)	3 (30%)	1 (11.11%)
Yes	28 (75.67%)	7 (58.33%)	6 (100%)	7 (70%)	8 (88.88%)
**Recurrence**					
No	31 (83.78%)	12 (100%)	5 (83.33%)	9 (90%)	4 (44.44%)
Yes	6 (16.21%)	0 (0%)	1 (16.66%)	1 (10%)	5 (55.55%)

BBT: benign bone tumor; MBT: malignant bone tumor; STT: soft tissue tumor.

**Figure 1:**
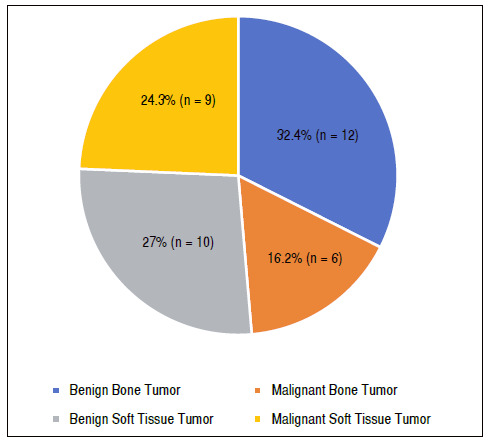
Prevalence of elbow injuries.

Figure 1 shows the prevalence of elbow injuries. A higher prevalence of benign bone tumors occurred, with 32.43% (n = 12) followed by benign soft tissue tumors, with 27.0% (n = 10). Table 2 shows the distribution of diagnoses of tumors that affected the elbow, with Synovial Sarcoma being the most prevalent (10.8% of cases).

**Table 2:** Distribution of elbow injury diagnoses.

	Total
	n (%)
	37 (100%)
**Benign bone tumor**	
Aneurysmal bone cyst	3 (8.1%)
Fibrous dysplasia	3 (8.1%)
Osteoid osteoma	3 (8.1%)
Charcot	1 (2.7%)
Solitary bone cyst	1 (2.7%)
Osteochondroma	1 (2.7%)
**Malignant bone tumor**	
Osteosarcoma	3 (8.1%)
Cavernous hemangioma	1 (2.7%)
Multiple myeloma	1 (2.7%)
Metastasis	1 (2.7%)
Ewing sarcoma	1 (2.7%)
**Benign STT**	
Lipoma	3 (8.1%)
Schwannoma	2 (5.4%)
Chondromatosis	1 (2.7%)
Fibromatosis	1 (2.7%)
Neurofibroma	1 (2.7%)
Inflammatory granuloma	1 (2.7%)
**Malignant STT**	
Synovial sarcoma	4 (10.8%)
Squamous cell carcinoma	1 (2.7%)
Malignant fibrohistiocytoma	1 (2.7%)
Lymphoma	1 (2.7%)
Liposarcoma	1 (2.7%)
Malignant PEComa	1 (2.7%)

STT: soft tissue tumor.

Table 1 also shows the results of prevalence and means of elbow lesions according to the variables studied.

## DISCUSSION

Bone and soft tissue tumors in the elbow are rare, with 37 tumors identified over a 40-year period in this case series. Relevant literature on the subject is sparse, for this reason this study brings relevant information to the area of studies on musculoskeletal tumors in the elbow region. Despite the rarity, it is important that the orthopedic surgeon knows the epidemiological profile and characteristics of the lesions so that they can properly care or refer the patient to the reference center in oncologic orthopedics.

Benign Bone Tumor was the most found lesion in this study; n = 12 (32.43%). The most common tumor in this series was Aneurysmal Bone Cyst, along with Osteoma Osteoid and Fibrous Dysplasia. This type of tumor was more present in adolescents and had a higher prevalence in cases of pathological fracture and second higher prevalence in pain. No case had lesion recurrence after undergoing the surgical procedure. These results are consonant with the study by Halai et al.^1^

Malignant Bone Tumors were more diagnosed in young adults (33.25 ± 26.5 years), with a similar prevalence among genders. Osteosarcoma was the most prevalent diagnosis in this type of tumor. These results agree with Halai et al.,^1^ who report in their study that most cases presented painful edema or mass detectable on physical examination, different from those of this study.

Benign Soft Tissue Tumor represented 27.02% of the cases in this study, with prevalence in women. In total, 70% of the cases showed complaint of pain and 80% of increased volume in the region. The most common diagnoses of this type of tumor were Lipoma (8.1%) and Schwannoma (5.4%).

When malignant soft tissue tumors were analyzed, a predominance in men, adults (mean age of 42 years) were observed, and a higher prevalence of the diagnosis of Synovial Sarcoma (10.8%). Recurrence rate was the highest among all types of tumors studied, 55.5%. Creighton et al.^9^ reported 10 patients with tumors in the elbow region in their series of 61 sarcomas of upper limb soft tissues. Correa-González et al.^5^ described that the elbow region usually has higher rates of residual disease and, thus, a higher incidence of local recurrence, due to the anatomical complexity of the elbow, which hinders resection with a wide oncological margin.

This study aimed to collect and define the epidemiological profile of patients with lesions in the elbow region. However, this study has limitations, such as the study design (case series) and data obtained from a single center, which limits the interpretation of the findings in the national context.

## CONCLUSION

The tumors that affect the elbow in our series are mainly benign tumors, of bone involvement or soft tissue. Knowing the epidemiological profile of elbow lesions can help to improve the understanding of the pathology and, consequently, therapeutic success. Early referral to a specialist is the fastest way to provide specialized multidisciplinary team care for these rare tumors.
